# Proving hydrogen addition mechanism from manure to coal surface obtained by GC-MS and ^1^H-NMR analysis

**DOI:** 10.1038/s41598-019-45254-4

**Published:** 2019-06-24

**Authors:** Cemil Koyunoğlu, Hüseyin Karaca

**Affiliations:** 1 Energy Institute, Istanbul Technical University, Ayazaga Campus, 34469, Istanbul, Turkey; 2 Chemical Engineering Department, Inonu University, 44280, Elazığ Road 15th km, Malatya, Turkey

**Keywords:** Chemical engineering, Asymmetric synthesis, Synthetic chemistry methodology, Surface chemistry

## Abstract

In this study, to explain the possibility of hydrogen transfer paths from manure to coal, Elbistan lignite (EL) combined with manure liquefaction of oil + gas products were analysed with Gas Chromatography-Mass Spectroscopy (GC-MS) and Nuclear Magnetic Resonance Spectroscopy (^1^H-NMR) technique. In the same way, it is observed that oils which as they fragment to an alkane-alkene mixture, serve as a hydrogen “sponge” and put a serious hydrogen need on the parts of the free radicals and molecules that are currently hydrogen poor. Concerning Elbistan lignite and manure do not have any aromatic hydrogen. Moreover, when the aromatic compounds were hydrogenated, their aromatic hydrogen was transformed to naphthenic hydrogen. Hydrogen transfer was due to isomerization of heptane from 3-methylhexane obtained in test oil where only manure was present as hydrogen donor in the liquefaction environment despite hydrogenation of isomerization from naphthalene to azulene.

## Introduction

**Figure 1:**
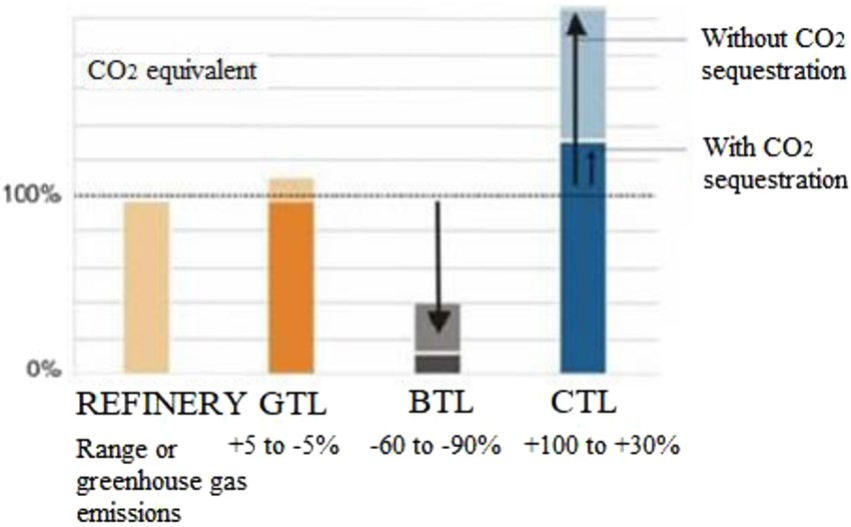
Well to wheel CO_2_ emissions of synthetic fuels in comparison to petroleum diesel.

To meet future demand in motor fuels, coal will play a key role in areas with large coal resources and lacking crude oils. Axens’ direct coal liquefaction (DCL) process is available to produce high-quality distillate fuels using commercially proven ebullated-bed reactor system. While indirect coal-to liquids (CTL) technologies are based on Fischer-Tropsch technology, both DCL and CTL plants should integrate carbon capture and storage (CCS) solutions owing to their higher well-to-wheel CO_2_ emissions (Fig. 1)^1–3^.

Alternative liquid fuels, i.e., first- and second-generation ethanol, biodiesel, gas-to-Iiquids (GTL), biomass-to-Iiquids (BTL), coal-to-Iiquids (CTL) and DCL, represent about 2.5% (energy content) of the on-road demand. It is estimated that they could represent up to around 7% in 2020 and 9–10% in 2030^4–6^.

Clean fuel projects are environmentally driven; thus, no additional revenue is anticipated. For such projects, minimizing the initial investment becomes a very important objective^7^. In this study the oil products of Elbistan lignite with manure which conducted in our previous study characterized by GC-MS technique in order to obtain substances for the clean fuel^8^.

The ^1^H-NMR technique of hydrogen transfer mechanism which is crucial for hydrocracking mechanism determination of oil products for co-liquefaction Elbistan Lignite combined with manure biomass is presented in this study. It has been showed in the present investigation that only the results of ^1^H-NMR analysis show that oils obtained with pyrolysis in combination with biomass of a low-rank coal pyrolysis only contain aliphatic compounds that are mainly linked to aliphatic compounds and have a lower percentage of the aliphatic compounds obtained. It has also been found that the amount of aromatic in the oils obtained in the pyrolysis experiments together increases^9^. GC-MS, as well as other GC techniques, are widely applied for the analysis of organic species in liquid products from coal or coal liquids, but the analytical data output is limited to the ionization mechanism and instrumental design. For unstable structures and non-volatile compounds with relatively high polarity thermal changes in coals with complex structure can be improved by solutions such as a series of atmospheric pressure, ionization techniques, and ambient ionization methods^10–13^. Examples of these unstable structures include naphthalene, this molecule obtained by the liquefaction method from coal can be detected by gas chromatography technique^14^. In addition, the two-dimension gas chromatography technique performed by another direct liquefaction method, as in this study, proves that the direct liquefaction method is more advantageous than the other liquefaction technique in terms of the selective product acquisition^15^.

### Understanding naphthalene to azulene reaction mechanism

As shown in Fig. 1, the stars around the benzene ring are, as a conjugated hydrocarbon molecule, when naturally given atoms at the end of each pair of bonds (in a counterclockwise rotation), if all the stellar atoms have only untranslated neighboring atoms and/or vice versa. The conjugate is called the hydrocarbon molecule alternative. For example, naphthalene is a conjugated hydrocarbon and is a conjugated hydrocarbon with no azulene alternative^16^.

**Figure 2:**
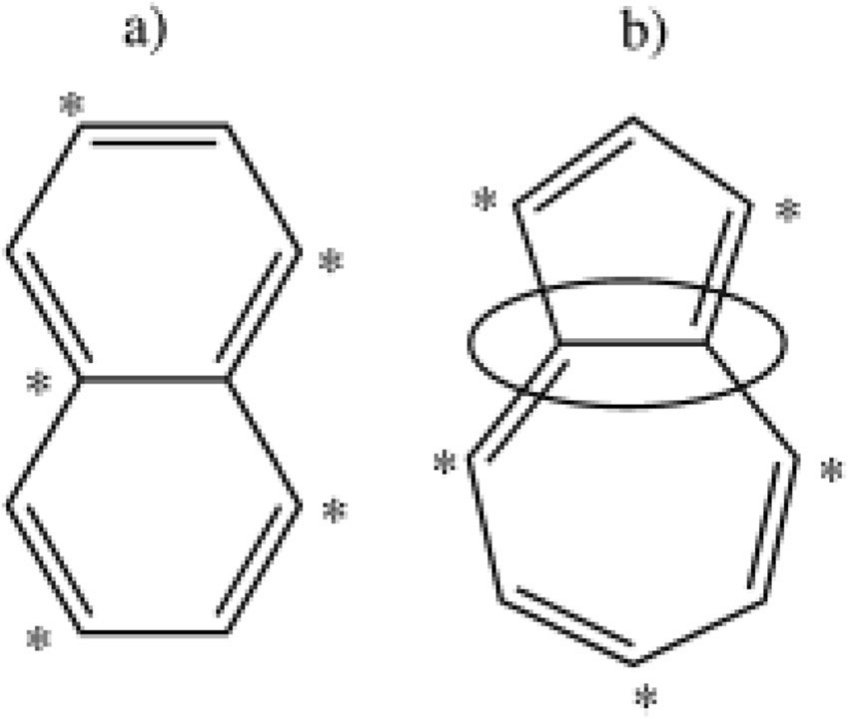
Structures of naphthalene (**a**) and azulene (**b**).

As described in Fig. 2, Gargurevich declared azulene produces as a R_5_R_4_R_5_H- type product with hydrogen elimination according to R_5_R_4_R_5_H- types which are isomerized to R_7_R_5_H- in the presence of H-catalysts at high reaction temperatures^17^. In the thesis study, energy barriers for the reactions seen in Fig. 2 were estimated using MNDO (Modified Neglect of Diatomic Overlap) methods. The formation temperature of R_5_R_4_R_5_H- is estimated to be 95 kcal/mole, which is considerably lower than the value reported in the synthesis studies for bi-radical intermediates^18^.

Thus, the sum of the reactions above mentioned is;

1 $${\rm{Naphthalene}}+{\rm{H}}\leftrightarrow {\rm{Azulene}}+{\rm{H}}$$

According to QRRK (Quantum Rice‐Ramsperger‐Kassel) theory the estimated reaction coefficient is k = 5.11 × 10^31^T^−4,192^ exp(−91.957/RT) cal, sec, cc, mole units in Ar bath, at 1.0 atm^18^.

Further evidence that the aforementioned reaction steps (see Fig. 2) provides a way to isomerization of naphthalene is the work of the researchers in 1993, including the addition of CH_2_ radicals to aromatics^18^.

The following study findings show that the hydrogen transfer to the coal surface without the use of hydrogen donor solvent (tetraline) from the manure added to the coal, both GC-MS (presence of azulene in the liquefaction products obtained from all optimum experiments when added to the coal, not in the presence of tetraline) and ^1^H-NMR results (proving the presence of aromatic compounds in the liquefaction product) and the fact that the naphthalene is converted to azulene due to the presence of hydrogen summarized by the literature above^18^.

### n-heptane to 3-methylhexane isomerization

3-methylhexane was one of the two main isomers during n-heptane isomerization^19^. Research indicates that this reaction occurs at nearly 200 °C which is under reaction temperature conducted in this study^19^. According to literature the progression of hydrogen addition reactions to heptane with less selectivity in alcohols and heptane mixtures compared to all other solvents^20^ has led us to conclude that the red mud with a catalytic effect in supercritical water conditions is not effective in this reaction^20^. Therefore, the transfer of hydrogen from the manure to the coal surface is very clear.

## Methods

### Materials

In this study, manure (collected from Malatya Sultansuyu Agriculture Operation Inc.) and lignite (gathered from Elbistan-Turkey) were brought to the laboratory and dried in an atmospheric conditions for 24 hours. The samples were crushed, ground and sieved to obtain a particle size of −20 mesh + 14 mesh, according to the results of the individual direct liquefaction experiments. Tetrahydrofuran, tetraline, n-hexane, toluene, ethylene glycol (EG), acetone and ethanol were purchased in analytical purity from Merck and Riedel-de Haen. An autoclave (PARR 500 ml stainless steel) was used for the liquefaction experiments. In our previous publications, the effect of different process parameters on liquefaction is presented in detail^6^. However, in this work, the coals belonging to the optimum process parameters determined as characterization were studied by 2 different characterization methods. The data pertaining to each analytic are described below.

### Characterization methods

In this study, GC-MS results were expected to expose EL with manure promote fuel differs. GC-MS results also were expected that co-liquefaction of EL products show petroleum fuel components and other analyses present EL combined with manure (catalyzed by red mud). GC-MS analyses conducted with Agilent Technologies 6890 N Network GC System model gas chromatography, and Agilent Technologies 5973 inert Mass Selective Detector mass spectrometer (Agilent Technologies, Santa Clara, CA) at the Inonu University Scientific and Technology Center Research laboratory. ^1^H-NMR results in assistance previous work were anticipated that red mud acts as a catalyst and manure were as hydrogen donor. ^1^H-NMR analyses conducted with Nuclear magnetic resonance spectroscopy (Bruker 300 MHz, Ultrashield, Bruker Corporations, Billerica, USA) at the Inonu University Scientific and Technology Center Research laboratory. GC-MS system analysis conditions are given in Table 1.

**Table 1:** GC-MS System analysis conditions.

Column	HP-INNOWAX
**Capillary nominal column**	
Film thickness	0.25 μm
Distance	60 m
Diameter	0.25 μm
The carrier gas (He)	5 ml/min
The amount of gas	1 µL
Dedector	FID
Dedector temperature	250 °C
Initial temperature	60 °C (1 min isothermal)
Final temperature	250 °C (10 min isothermal)
Heating rate	25 °C/min
Solvent	C_6_H_14_

### Liquefaction method

**Figure 3:**
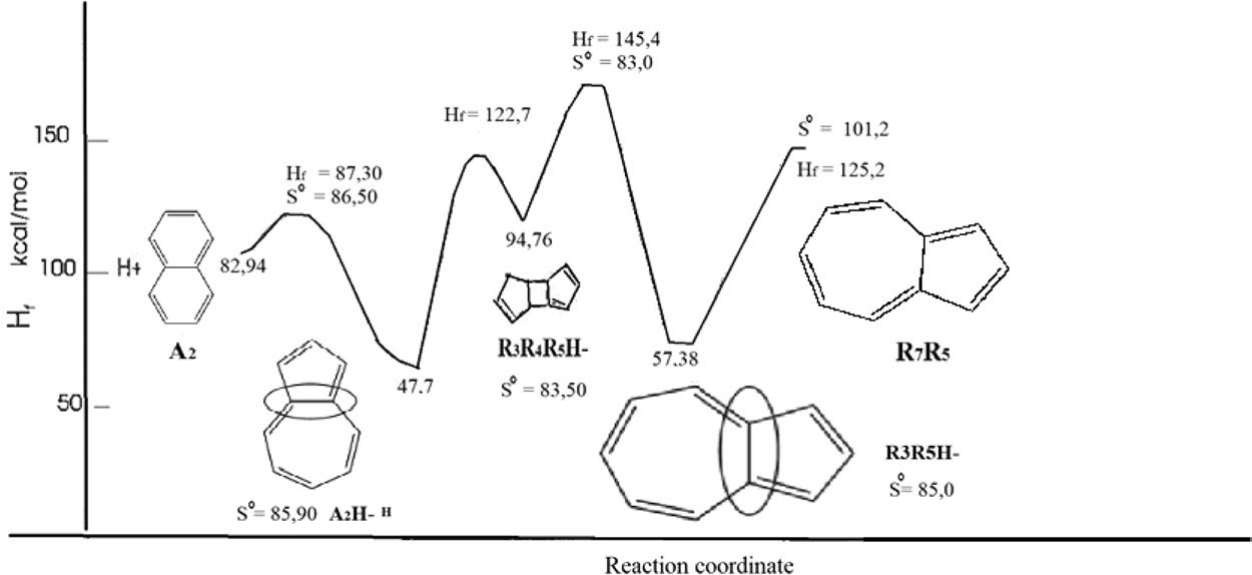
Energy diagram for H-radical catalysis of naphthalene isomerization to azulene.

Lignite was chosen to investigate the effects of different mixing ratios, manure ratio, oil yield and chemical properties of the products (char, asphaltene, and preasphaltene). Lignite and manure (total weight 30 g) were sealed and mixed with 90 ml of tetraline without catalyst to make the feedstock slurry in the autoclave which had been cleaned with inert gas (nitrogen). In this study, tetraline was used as solvent. The initial pressure was set to 20 bar and then the reactor was heated to a temperature of 400 °C. After being programmed for mixing and allowed to stand for 60 minutes. After the experiments, a water/ice bath was used to cool the reactor to room temperature. After the gas product was removed from the system, the reactor was opened and the products were transferred to a soxhlet extract with tetrahydrofuran (THF). In this research, the liquefaction products were divided into two, dissolved and not soluble in THF (see Fig. 3), and the resulting char was extracted with acetone and then dried by leaving vacuum at 80 °C for 24 hours. Extractable fractional oil (n-hexane-extractable fraction), asphaltene (hexane non-extractable fraction) and preasphaltene (toluene-extractable fraction) with THF. The oil samples were separated into sample containers for GC-MS and ^1^H-NMR analyses of the optimum parameters determined by the percentage of oil + gas and total conversion calculated as mass percentages.

## Results and Discussion

### GC-MS analysis

Some recent researches on gas chromatography of liquefied oil products have found that compounds can be classified as alkanes, alkenes, phenols, aromatic hydrocarbons, and some other oxygen-containing compounds, when investigating lignite valuable liquid products, the liquid products are very complex^21–26^. The results of the studies show that there is a lot of oxygen in the oil consists mainly alcohols, acids, ketones, esters and aldehydes. The complexity of the sample in the analysis makes it difficult to determine the practical use of oils obtained from coal fuels as liquid fuel. Therefore, in order to reduce the complexity of the liquid product obtained in this study, the highest total conversion in the liquefaction experiments, and in particular the experiments in which the oil + gas conversion was obtained, were determined and the oil samples were analyzed in gas chromatography^27^.

**Figure 4:**
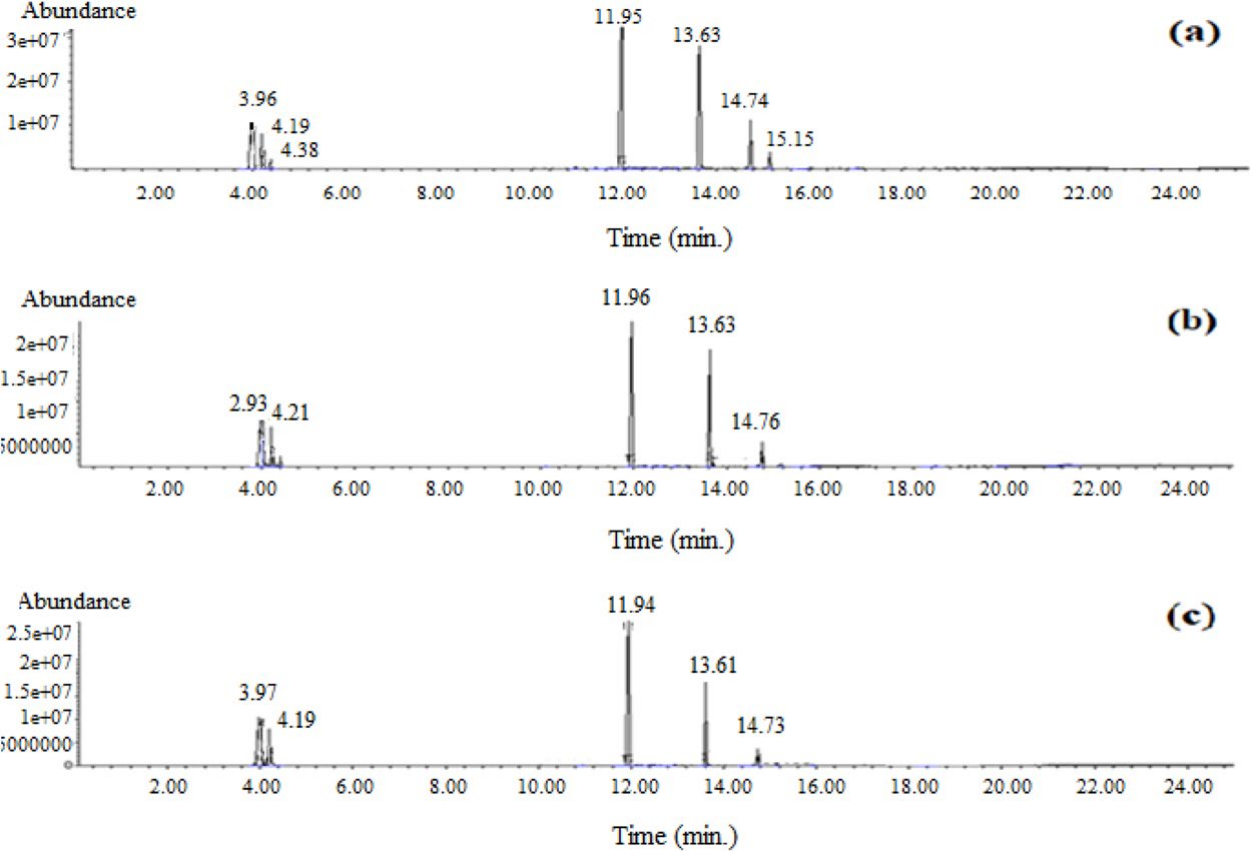
GC-MS chromatograms of oils (**a**: E1, **b**: E6, **c**: E12).

In this research, in the experiments chosen based upon the greatest total and oil + gas conversion, the analyses are shown in Table 1 and the associated chromatograms offer in Fig 2(a–c), for the oils and derivatives, azulene, obtained as a result of the liquefaction procedure performed under various conditions. Likewise, the GC-MS analysis results are provided in Table 2. Firstly, as it can be observe from Tables (2–5) and Fig. 4(a–c) (Experiment number 12 is the conditions of alone EL liquefaction), the oils acquired by the liquefaction of the Elbistan lignite and manure, under catalytic conditions, using C_10_H_12_ as a solvent, are mainly made up of compounds such as straight chain hydrocarbons like C_10_H_8_ and derivatives. Secondly, C_10_H_12_ and derivatives, C_6_H_6_ and derivatives, (C_6_H_14_, C_10_H_8_), methyl-cyclopentane (C_6_H_12_), butylated hydroxytoluene (C_15_H_24_O). Upon changing the liquefaction conditions, a considerable change has not been observed the composition of the oils.

**Table 2:** Compounds from the one of the optimum experiments (Experiment Number:1) (% Abundance > 1.00).

Amount in total*	Similarity %	Probable compound
14,06	86	Hexane
37,12	97	1,2,3,4-tetrahydronaphthalene
25,79	87	Azulene or Naphthalene
6,76	97	Butylated hydroxytoluene
4,81	90	Methyl-cyclopentane
2,22	95	2-methyl-naphthalene
1,09	91	Cyclohexane
0,64	91	2,3-dihydro-4-propyl-1H-ındene
0,60	96	2,6,10,14-tetramethyl Pentadecane
0,55	96	1-ethyl-naphthalene
0,50	95	1,4-Dihydronaphthalene
0,46	90	(2-methyl-1-butenyl)-benzene
0,44	97	1,2,3,4-tetrahydro-1-methyl-naphthalene
0,41	93	1-propyl-naphthalene
0,30	95	Heptadecane
0,27	96	1-ethyl-naphthalene
0,22	91	1-ethenyl-3-ethyl-benzene
0,17	99	Tetracosane
0,17	87	1-Phenylethynyl cyclohex-1-ene
0,16	97	Pentadecane
0,16	96	2-ethyl-1,2,3,4-tetrahydro-naphthalene
0,15	99	Tricosane
0,14	95	2,3,6-trimethyl-phenol
0,13	90	3,4-dihydro-1(2 H)-naphthalenone
0,11	93	4-ethyl-phenol

*(abundance, %).

**Table 3:** Compounds from the one of the optimum experiments (Experiment Number:6) (% Abundance > 1.00).

Amount in total*	Similarity %	Probable compound
3,76	86	Hexane
5,67	91	Methyl-cyclopentane
36,25	96	1,2,3,4-tetrahydronaphthalene
1,43	91	Cyclohexane
19,50	94	Azulene
3,78	86	Butylated hydroxytoluene
1,96	96	Eicosane
2,69	89	Eicosane
1,52	90	Docosane or Nonadecane
1,84	91	Nonahexacontanoic acid
1,43	91	Cyclohexane
0,92	90	Heptane
0,48	90	(1-ethyl-1-propenyl)-benzene
0,42	97	1,2,3,4-tetrahydro-1-methyl-naphthalene
0,54	97	2,3-dihydro-4,7-dimethyl-1H-ındene
0,57	91	2,3-dihydro-4-propyl-1H-ındene
0,55	94	1,2,3,4-tetrahydro-1-propyl-naphthalene
0,17	87	1,2,3,4-tetrahydro-1-naphthalene
0,97	95	2-methyl-naphthalene
0,15	96	1-ethyl-naphthalene
0,29	95	1,1a,6,6a-tetrahydro-cycloprop[a]indene
0,22	91	2,4,6-trimethyl-phenol
0,23	96	1-ethyl-naphthalene
0,26	94	3,4-dihydro-1(2 H)-naphthalenone
0,22	95	Pentacosane
0,13	98	1,2,3,4-tetrahydro-6- methyl-naphthalene
0,13	93	1,2,3,4-tetrahydro-5-methyl-naphthalene
0,13	96	Tridecane

**Table 4:** Compounds from the one of the optimum experiments (Experiment Number :8) (% Abundance > 1.00).

Amount in total*	Similarity %	Probable compound
39,61	90	3-methyl-hexane
30,32	86	Hexane
23,98	90	Methyl-cyclopentane
5,30	91	Cyclohexane
0,36	96	1,2,3,4-tetrahydro-naphthalene
0,44	98	Butylated hydroxytoluene

**Table 5:** Compounds from the one of the optimum experiments (Experiment Number :11) (% Abundance > 1.00).

Amount in total*	Similarity %	Probable compound
23,01	86	Hexane
6,23	90	Methyl-cyclopentane
1,18	90	Heptane
1,57	91	Cyclohexane
44,36	96	1,2,3,4-tetrahydronaphthalene
15,96	94	Azulene
3,23	86	Butylated hydroxytoluene
0,14	91	1,3-dimethyl-cyclopentane
0,34	93	(2-methyl-1-butenyl)-benzene
0,31	97	1,2,3,4-tetrahydro-1-naphthalene
0,34	97	2,3-dihydro-1H-ındene
0,38	95	1,4-dihydronaphthalene
0,45	94	1-Ethyl-1,2,3,4-tetrahydronaphthalene
0,41	94	1,2,3,4-tetrahydro-1-propyl-naphthalene
0,19	90	1,2,3,4-tetrahydro-1-propyl-naphthalene
0,19	96	1-ethyl-naphthalene
0,25	95	3,4-dihydro-1(2 H)-naphthalenone

“In the Tables (2–5), it is seen that coal 3% red mud has 37.12% more tetraline, 25.79% more azulen and 37.09% other compounds are more abundant.” When increasing of red mud concentration was high with 9% the most abundant compound due to the analysis is 36.25% of tetraline, 19.50% of azulene, and the rest compounds with 44.25%. In our previous study, we qualitatively showed possible hydrogen transfer compared with hydrogen donor solvent (tetraline) with non-hydrogen donor solvents (distilled water) from manure to EL^3^. When obtaining GC-MS data from those experiments, 3-methyl-hexane with 39.61%, hexane with 30.32%, methyl-cyclopentane with 23.98% abundance, and cyclohexane with 5.30% abundance resulted. When obtaining 1/1 mixing rate between EL and Manure (the highest ratio) in the experiments, 44.36% of tetraline, 23.01% hexane, 15.96% of azulene, and the rest compounds with 16.67% abundance collected in the oil products. Azulene (C_10_H_8_) known as being the valence isomer of naphthalene^28^. Azulene (C_10_H_8_) is a stable isomer of naphthalene-containing a five-member and a seven-member ring^17,28,29^. The fact that the azulene is located in the manure of farm animals fed with the herbs of vegetable origin can be explained by the obtaining of the azulene in the gas chromatography analysis. For example, Ma *et al*. obtained the azulene obtained by SD method in schisandra chinensis baill fruits with an abundance of 2.561%, and the azulene obtained by HD method was 0.554%^30^. Kovatz found the azulene compound with 0.2% abundance in rose oil^31^. Zeng *et al*. Liquefied the beech wood using solar energy and determined the azulene molecule in the liquid product obtained under 600 °C heating condition^32^. He *et al*. found that they had obtained azulene compound as a result of GC-MS analysis in 11.76 minutes of bio-oil obtained by liquefying the mallee leaves in the fluidized-bed reactor^33^. In another study, Spitellar and Jovanovic showed the azulene compound in the vaporization of naphtha by steam pyrolysis by GC characterization^28^. In view of the fact that azulene and naphthalene are 12.8 and 10.928 minutes in the literature due to the results of gas chromatography, in Fig. 3, it is seen that the azulene of 13.6 minutes in each of the three graphs is closer to the pure azulene^34,35^. The fact that the formation enthalpy of the azulene is less than the naphthalene in the thermodynamically presents the fact that the azulene is formed by the heat transfer to the naphthalene during the reaction^36–38^. In the benzene ring, bond breaks during the formation of azulene formed by binding 1 carbon to the other benzene ring of the naphthalene to the other benzene ring are also considered to be endothermic and in many cases the chain is transferred to the monomer. The reaction proceeds with a hydrogen transfer or hydrogen abstraction reaction between the increased radical and the monomer molecule highlighted^39–41^.

Finally, the primary point of the GC-MS outcomes is the some obtained compound from co-liquefaction experiments rather than only one component (e.g. only coal) liquefaction experiments consisted of some petroleum fuels, for circumstances when lignite liquefied with manure the obtained compound which is eicosane is the compound in jet fuel, kerosene, and diesel fuel^42^. Nevertheless, docosane and nonadecane has been discovered in diesel fuels^43^. Additionally, nonahexacontanoic acid was found in a grease^44^. The outcomes state alternative production of petroleum components instead of oil refining, and it is the solution of low-cost oil production which is dependent on importing oil.

### ^1^H-NMR analysis

Today, researchers have generally used the ^1^H-NMR technique to measure the relaxation properties of water in coal formations. In these studies, the majority of the water associated with low-rank coal was found to be on the coal surface above 0 °C but not surface. In some investigators, it has been found that water has hydroxyl-free compounds in free form, in pores, or in three forms with three different expansions, that is, in free form or in non-free form, even in coal pores. In this study, the ^1^H-NMR technique was used to show the presence of hydrogen transfer to the coal surface when liquefied with a hydrogen donor biomass (farm manure) with coal^6,45^.

**Figure 5:**
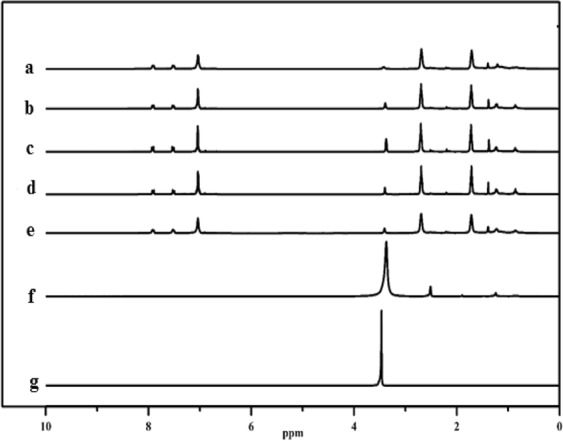
H-NMR analyses of the optimum process parameters: (a) E4, (b) E13, (c) E19, (d) E20, (e) E11, (f) M, (g) EL.

In this study, the ^1^H-NMR spectra of the chosen heavy oil constituents obtained from the liquefaction experiments and that manure and Elbistan lignite (EL) samples were examined, and shown in Fig. 5. For this purpose, ^1^H-NMR can be used to identify the contents of aromatic hydrogen, by contrast, it can not provide the distribution of aromatic ring number^46,47^. First of all, the ratio of the aromatic hydrogen (Experiment numbers of 4 (non-catalytic conditions), 11 (catalytic conditions), 13 (EL/Manure ratio of 1/1, w/w), 19 (reaction temperature of 350 °C) and 20 (reaction temperature of 400 °C)) to the total hydrogen was identified by the ratio of the incorporated peak area varying from 6.5 to 8.5 ppm to that of the total peak area in the ^1^H-NMR spectrum. The second, the oils ratio of the aromatic hydrogen to the total hydrogen from 27.8% to 31.8%. However, when no catalyst uses in the co-liquefaction (Experiment number 4) the aromatic hydrogen ratio is 31.8%, also, catalyst usage of co-liquefaction (Experiment number 11) the aromatic hydrogen ratio has to do with 27.8%. The third, aliphatic hydrogen content and aromatic ring substitution index increase with the red mud, particularly for H_α_ proton, however, aromatic hydrogen percentage is decreased. It is because aliphatic components concentrate in the alkane-alkane mixture which has similarly low aromaticity. In the same way, it is observed that oils which as they fragment to an alkane-alkene mixture, serve as a hydrogen “sponge” and put a serious hydrogen need on the parts of the free radicals that are currently hydrogen poor. As for it can state that catalyst promotes H transfer to EL surface area during co-liquefaction.

## Conclusions

With regard to GC/MS analysis, it can be said that the liquefaction of coal with manure instead of liquefaction only increases fuel variables, such as obtaining a new component for jet fuel, kerosene, diesel and grease. These components nowadays show that, instead of producing low-quality lignite, it has the ability to liquefy the manure, which is catalyzed by the red mud in the production of alternative fuels and raw materials to petroleum to spread the right energy strategy. This result also confirms the hydrogen transfer of the manure to the EL. However, the increase in the rate of manure entering the liquefaction resulted in an increase in the aliphatic hydrogen content. In other words, when more manures are used, hydrogen transfer from manure to coal increases. Thus, the manure used as a component in the liquefaction prior to this operation functions as a hydrogen donor. Because Elbistan lignite and manure does not have any aromatic hydrogen. Further, when the aromatic compounds are hydrogenated, the aromatic hydrogen is converted to naphthenic hydrogen.

In this study, we have used gas chromatography-mass spectrometry technique to qualitatively prove the hydrogen transfer mechanism we have shown in our previous studies. Gas chromatography-mass transfer analysis of the gas transfer mechanism of the tetraline liquefaction solvent known as hydrogen carrier in the literature to the coal has not been described up to now. However, in the literature with this work is described for the first time by the radical conversion of the benzene ring by hydrogen transfer to the monomer molecule first described as a result of the isomerization reaction from naphthalene (by heat transfer from the reaction medium). The organic (hydrogen-carrier) solvents used in the liquefaction are generally used to form the active site on the coal surfaces. The solvent is then completely recovered from the final products. This reaction mechanism is an example for clean production applications, especially in the chemical industry. It is ideal, sustainable. Because as a renewable energy source, as a biomass hydrogen carrier, it will reduce the energy consumption of liquefying due to not using organic solvents by opening the way for solventless production applications with only coal + biomass liquefaction option.

The presence of aromatic hydrogen was determined in all experiments by ^1^H-NMR technical analysis. According to GC-MS analysis of the oil obtained in the test results, only the manure is given as hydrogen donor component in the coal liquefaction medium, and in other experiments, tetraline as hydrogen donor, abundance of 20% in all the oil values in the presence of tetraline using only the manure of the azulene compound. The highest abundance of naphthalene was obtained in the experiment, but the most abundant 3-methyl hexane as a result of isomerization of heptane clearly demonstrated its transfer from hydrogen manure to coal radicals.
